# CD74 in Apoptotic Macrophages Is Associated with Inflammation, Plaque Progression and Clinical Manifestations in Human Atherosclerotic Lesions

**DOI:** 10.3390/metabo12010054

**Published:** 2022-01-10

**Authors:** Wei Li, Nargis Sultana, Linda Yuan, Claes Forssell, Xi-Ming Yuan

**Affiliations:** 1 Obstetrics and Gynecology, Department of Biomedical and Clinical Sciences, Linköping University, 581 85 Linkoping, Sweden; 2 Occupational and Environmental Medicine, Department of Health, Medicine and Caring Sciences, Linköping University, 581 85 Linkoping, Sweden; Nargis.Sultana@regionostergotland.se (N.S.); li8571yu-s@student.lu.se (L.Y.); ximing.yuan@liu.se (X.-M.Y.); 3 Laboratory Medicine, Linköping University Hospital, 581 85 Linkoping, Sweden; 4 Vascular Surgery, Linköping University Hospital, 581 85 Linkoping, Sweden; clafors@gmail.com

**Keywords:** apoptosis, atherosclerosis, CD74 (cluster of differentiation 74), macrophage, thrombosis, plaque rupture

## Abstract

The aim of this study was to investigate whether CD74 levels in atherosclerotic lesions are associated with inflammation, apoptosis, plaque severity, and clinical symptoms among patients with carotid atherosclerosis. We further studied whether CD74 expression is associated with apoptosis in macrophages induced by 7ketocholesterol (7keto). Sixty-one carotid samples (39 males and 22 females) were immunostained with macrophages, smooth muscle cells, CD74, ferritin, TUNEL (Terminal deoxynucleotidyl transferase dUTP nick end labeling), and thrombin receptors. Double immunocytochemistry of CD74 and caspase 3 or CD74 and Annexin V was performed on THP-1 macrophages exposed to 7keto. In human carotid plaques, CD74 expression is lesion-dependently increased and is associated with necrotic core formation and plaque rupture, clinical symptoms, macrophage apoptosis, ferritin, and thrombin receptors. CD74 levels were inversely correlated to high-density lipoproteins and statin treatment, and positively correlated to triglycerides. In THP-1 macrophages, 7keto induced a significant increase in levels of CD74, ferritin, and apoptotic cell death. This study suggests that CD74 in apoptotic macrophages is linked to inflammation and thrombosis in progression of human atherosclerotic plaques, lipid metabolism, and clinical manifestation in atherosclerosis. Surface CD74 in apoptotic macrophages and ferritin production induced by oxidized lipids may contribute to inflammation and plaque vulnerability in atherosclerosis.

## 1. Introduction

Atherosclerosis is an inflammatory disease that involves cross-talk between shared pathways involved in adaptive and innate immunity [1]. Atherosclerotic lesions contain large numbers of immune cells, especially macrophages and T cells that are important in initiating inflammatory responses. In atherosclerosis, macrophages produce proinflammatory cytokines that participate in lipid retention, cell death, formation of a necrotic core, plaque instability, and thrombosis [1].

Cell death, inflammatory cytokines, and lipid accumulation are inextricably linked in atherogenesis [1]. Accumulated oxysterols in atherosclerotic lesions have several important pathological effects, including the induction of arterial cell death, amongst their diverse immunoregulatory roles [2]. Many studies have established the pro-apoptotic potential of several major oxysterols in atherosclerosis [3,4]. Oxysterols, particularly 7β-hydroxycholesterol and 7ketocholesterol (7keto), increase intracellular levels of reactive oxygen species (ROS) and induce apoptosis through the activation of death receptor-dependent (extrinsic) and mitochondrial (intrinsic) pathways. Our previous studies have demonstrated that oxysterol-induced apoptotic cell death is initiated by lysosomal membrane permeabilization (LMP) followed by increases in cellular oxidative stress and ferritin accumulation [5]. Ferritin, an iron storage protein, is considered an inflammatory marker in atherosclerosis [6]. Moreover, exposure of cells to 7-oxysterols switches macrophage phenotypes towards the pro-inflammatory profile of M1 macrophages [7].

Cluster of differentiation (CD) 74, also known as invariant chain, is a non-polymorphic type II integral membrane protein. The main function of CD74 is to regulate the trafficking of class II major histocompatibility complex (MHC) proteins in antigen-presenting cells. Surface expression of CD74 may also play a role as a receptor independent of MHC class II, and has been shown to act as a receptor to different cytokines in inflammatory disorders. Moreover, CD74 activation can mediate upregulation of TNF-related apoptosis-inducing ligand (TRAIL) leading to apoptotic cell death in human diabetic nephropathy [8]. However, whether CD74 expression is associated with apoptosis in atherosclerosis has not yet been studied.

It has previously been reported that the expression of CD74 is increased in human atherosclerotic plaques and plays a vital role in the inflammatory cascade during atherogenesis [9]. It has also been reported that deficiency of CD74 reduces atherosclerosis in mice [10]. However, it is unknown whether CD74 expression contributes to atheroma plaque progression and clinical symptoms, and whether an atheroma relevant oxysterol influences CD74 expression in macrophage apoptosis.

The aim of this study was to determine whether CD74 expression is associated with clinical symptoms or plaque severity among patients with carotid atherosclerosis. This study further investigated whether CD74 expression is related to apoptosis in human carotid atheroma and in macrophage apoptosis induced by 7keto, an atheroma relevant oxysterol that has been implicated in chronic inflammation of atherosclerosis [11].

## 2. Results

### 2.1. CD74 Expression Is Significantly Increased with the Progression of Human Atherosclerotic Plaques

**Figure 1:**
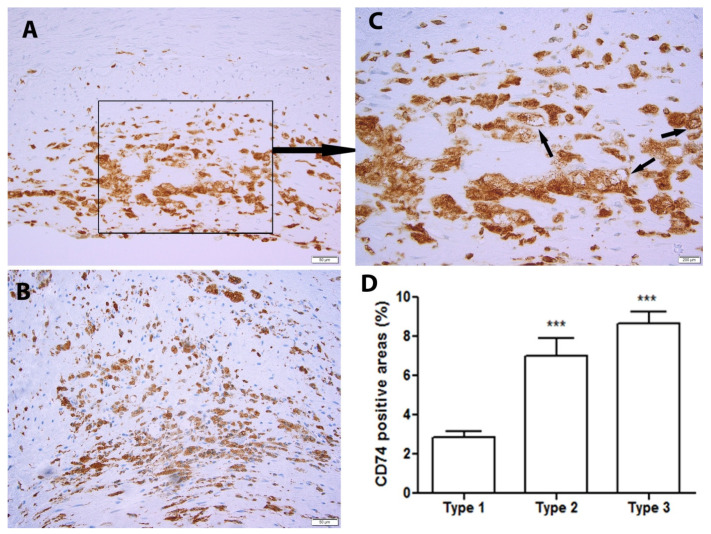
CD74 levels are significantly related to the severity of the atheroma plaque. Representative images of type 1 lesions (**A**), the squared area is shown in C with higher magnification and type 2 lesions (**B**). (**C**) An enlarged area from A indicate cell surface expression of CD74 (arrows). Image analysis of CD74-positive areas in type 1 (*n* = 47), type 2 (*n* = 33), and type 3 (*n* = 59) plaques (**D**). *** *p* < 0.001 vs. type 1 plaques.

We first assessed the presence of CD74 in human atherosclerotic plaques. CD74-positive staining was observed in nearly all of the samples (60/61). The plaques were then classified into type 1–3 as discribed in the Methods section, and the CD74-positive area in the plaques was further evaluated according to the classification. Increased CD74 levels were found more frequently in type 2 than in type 1 plaques (Figure 1A,B). Moreover, CD74 was predominantly expressed intracellularly, with partial surface expression (Figure 1C, arrows). Overall, CD74 was significantly increased in type 2 and type 3 plaques compared to type 1 plaques (Figure 1D).

### 2.2. CD74 Expression in Human Carotid Atheroma Is Significantly Associated with an Accumulation of Macrophages, Apoptosis, Ferritin, and Thrombin Receptor (TR)

**Figure 2:**
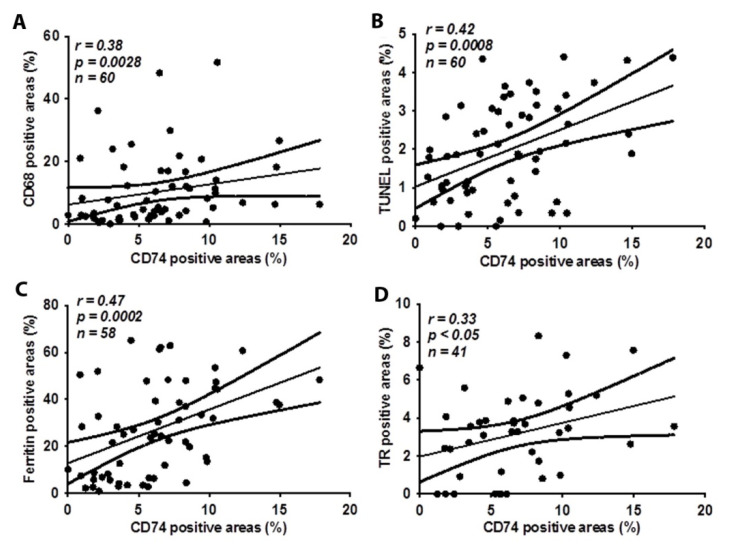
CD74 levels are positively correlated to CD68 positive macrophages, apoptosis, ferritin, and thrombin receptor (TR). Serial sections of carotid atherosclerotic lesions were stained with CD74, CD68, TUNEL, ferritin and TR. The correlation between CD74 and CD68 (**A**), TUNEL (**B**), ferritin (**C**), and the TR (**D**) were assessed by Spearman’s correlation coefficient test.

**Figure 3:**
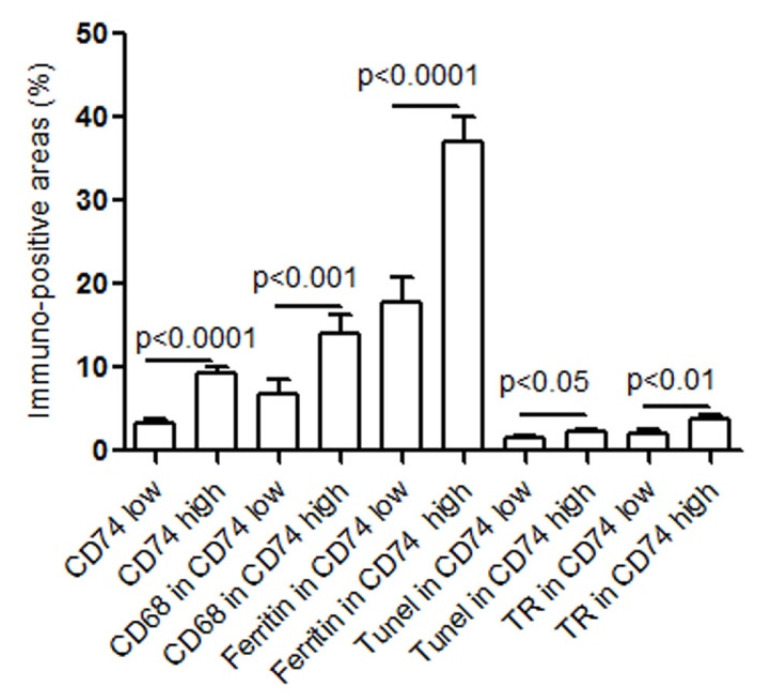
Lesions with higher levels of CD74 contain higher levels of macrophages, apoptosis, ferritin, and thrombin receptor. The levels of CD74 in the lesions were divided in two groups by the median value. The CD74 levels ≤ median value was defined as CD74 low (*n* = 31), while >median value was defined as CD74 high (*n* = 30). The CD68, ferritin, TUNEL, and TR levels were further divided in two groups according to CD74 low or high for comparison.

We have previously shown that accumulated macrophages in human atherosclerotic lesions contribute to necrotic core formation and plaque rupture [12,13]. Here, in an investigation of CD74 expression in relation to macrophages and SMCs in human carotid atheroma, we found that CD74 expression was significantly correlated to levels of CD68 positive macrophages (Figure 2A). The analysis of serial cross-sections from carotid atherosclerotic lesions revealed no correlation between CD74- and SMC actin-positive areas (r = 0.03, *p* = 0.81). Furthermore, CD74 expression was significantly correlated to apoptosis, as indicated by TUNEL positive areas (Figure 2B) and ferritin expression (Figure 2C). Moreover, CD74 was positively correlated with thrombin receptor (Figure 2D), a key player in mediating the interplay between coagulation and inflammation. The associations of CD74 with CD68 macrophages, ferritin, apoptosis (TUNEL), and thrombin receptor were further analyzed by dividing levels of CD74 into a low or high expression group using CD74 median value, namely CD74 low (≤median) group and CD74 high (>median) group. As shown in Figure 3, the levels of CD68, ferritin, TUNEL, and thrombin receptor were significantly higher in CD74 high expression group. Immunohistochemistry images of CD68, CD74, and TR in an atherosclerotic lesion on serial sections are shown in Supplementary Figure S1.

### 2.3. CD74 Expression Is Significantly Increased in Lesions from Symptomatic Patients and Related to Carotid Stenosis

**Figure 4:**
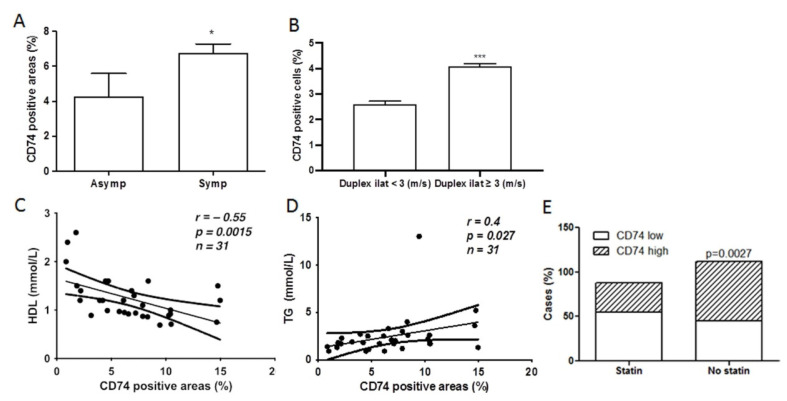
CD74 levels are significantly associated with clinical manifestations and serum TG, and inversely associated with HDL and statin treatment. (**A**) CD74 levels in symptomatic (Symp, *n* = 53) and asymptomatic (Asym, *n* = 8) patients. * *p* < 0.05 vs. asymptomatic group. (**B**) Comparatively higher levels of CD74 were seen in patients with severe carotid stenosis. *** *p* < 0.001 vs. duplex scan < 3 (m/s). (**C**,**D**) CD74 is inversely correlated to HDL levels (**C**) and positively correlated to TG (**D**). (**E**) Carotid plaques from patients without statin treatment showed higher levels of CD74 compared to those from patients who received statin treatment.

Next, we investigated whether CD74 expression in the lesions was related to the clinical symptoms of patients with carotid atherosclerosis. There was a significant increase in the CD74-positive area in lesions in symptomatic patients compared to asymptomatic patients (Figure 4A). Plaque types between groups of patients with symptoms or without symptoms were further analyzed. The results showed that 60% (12/20) of the plaques in the asymptomatic group were type 1, while 29.3% (35/119) of the plaques in the symptomatic group were type 1 (*p* < 0.05). According to carotid duplex scans, the samples were further divided into two groups (<3 m/s or ≥3 m/s) to examine a possible relationship between CD74 expression and the degree of carotid artery stenosis. Compared to patients with mild carotid artery stenosis, the CD74 immuno-positive areas were elevated in the groups with duplex scans ≥ 3 m/s (Figure 4B), indicating an association between CD74 expression and carotid atherosclerotic stenosis. Furthermore, the percentage of less severe stenoses (duplex scans < 3 m/s) was 25% in asymptomatic patients (2 of 8 patients) and 5.45% in symptomatic patients (3 of 55 patients).

### 2.4. CD74 Levels in Atherosclerotic Lesions Is Inversely Related to High Density Lipoprotein (HDL) Levels and Statin Treatment, and Positively Related to Triglyceride (TG) Levels

Increased CD74 expression contributes to inflammatory responses and ischemic stroke [14]. HDL has anti-atherosclerotic activity through various mechanisms, including anti-inflammatory functions [15]. In the present study, we found that CD74 expression was inversely correlated to plasma levels of HDL and positively correlated to TG (Figure 4C,D). Patients with higher levels of CD74 often received no statin treatment (Figure 4E). CD74 levels were higher in the plaques from males compared to those from females, without statistical significance (6.91 ± 0.61 and 5.26 ± 0.82, respectively).

### 2.5. CD74 Is Significantly Increased in Macrophages Exposed to 7keto

**Figure 5:**
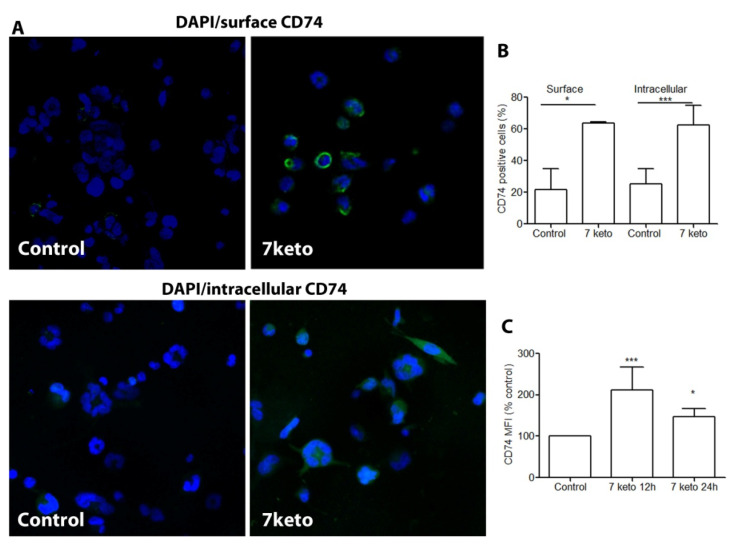
CD74 is increasingly expressed on the cell surface and intracellular space in THP-1 macrophages after exposure to 7keto. THP-1 macrophages were either left untreated or were treated with 7keto for 12 or 24 h. Following CD74 immunocytochemistry, separately for surface (no fixation and permeabilization) and intracellular expression, the cells were examined by confocal microscopy. (**A**) Representative photographs of surface and intracellular CD74. (**B**) Percentage of CD74 positive cells of surface (*n* = 2) and intracellular levels (*n* = 5) after 24 h. Data are mean ± SD. (**C**) CD74 mean fluorescence intensity (MFI,% of controls). Data are mean ± SD, *n* = 5. * *p* < 0.05 and *** *p* < 0.001 vs. control.

The accumulation of 7keto in oxidized lipoprotein deposits and atherosclerotic lesions is known to be involved in macrophage foam cell formation and atherosclerosis, and results in complex and potent inflammatory responses [2]. However, it is unknown whether 7keto induces CD74 expression in macrophages. Compared to untreated control cells, exposure to 7keto increased CD74 expression both on the cell surface and intracellularly (Figure 5A–C), both in terms of the percentage of positive cells and mean fluorescence intensity (Figure 5B,C).

### 2.6. Increased CD74 Is Significantly Associated with 7keto-Induced Apoptosis in Macrophages

**Figure 6:**
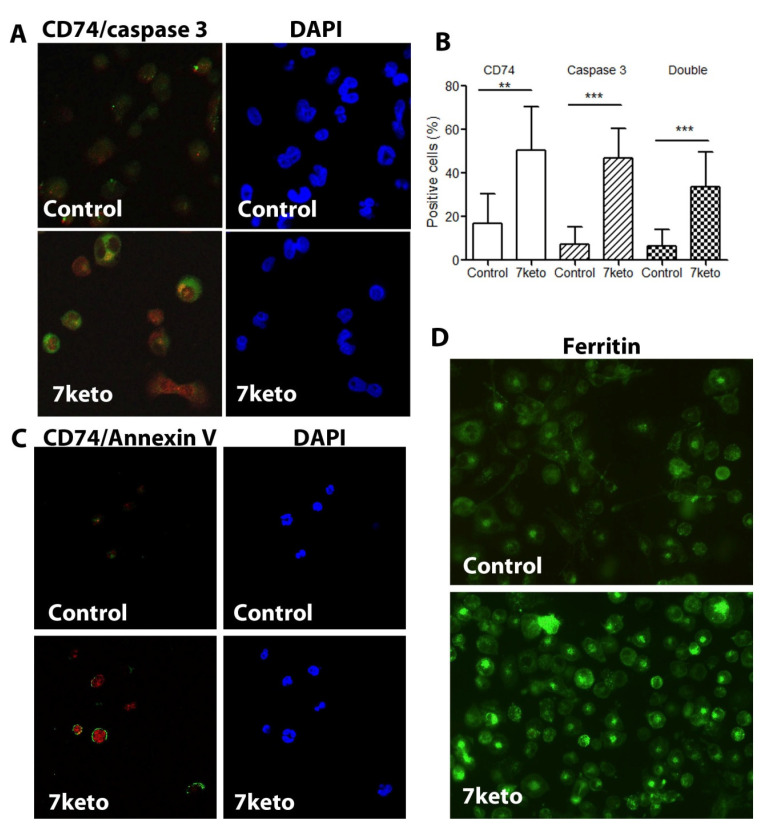
Increased CD74 following 7keto exposure is associated with increased ferritin and apoptotic cell death. THP-1 macrophages were either untreated or treated with 7keto for 24 h. Following double immunocytochemistry for intracellular CD74 and active caspase 3, the cells were examined by confocal microscopy. The percentage of positive cells for CD74, caspase 3, and CD74 with caspase 3 were counted. (**A**) Representative photographs of CD74 (red) and caspase 3 (green). Nuclei stained blue with DAPI. (**B**) Positive cells with intracellular CD74 and caspase 3 (%). Data are mean ± SD, *n* = 8–9. ** *p* < 0.01 and *** *p* < 0.001. (**C**) Representative photographs of CD74 (red) and Annexin V (green) in control and 7keto-treated cells. Nuclei stained blue with DAPI. (**D**) Representative photographs of ferritin immunocytochemistry in control cells and 7keto-treated cells.

As one of the major cytotoxic oxysterols, 7keto activates inflammation and induces cell death and apoptosis in different cell types [2,3,4,5]. Here, we examined the association between CD74 levels and apoptosis in our established cell model, 7keto-treated THP-1 macrophages. We found that 7keto treatment resulted in activation of caspase 3 and increased CD74 levels (Figure 6A). Quantitative analysis of the cells confirmed the significant increase in CD74 expression as well as activation of caspase 3 following 7keto exposure, in which about 50% cells expressed both CD74 and caspase 3 (Figure 6B). Compared to control cells, a significantly higher percentage of apoptotic cells showed increased CD74 levels (Figure 6B, double positive cells). Apoptotic cells induced by 7keto were further verified by Annexin V staining, in which cell surface expression of phosphatidylserine was identified and co-expression of Annexin V and CD74 was evident (Figure 6C). Moreover, 7keto induced apoptotic cell death was further confirmed by means of morphological analysis and flow cytometry (Figure S2). In the cell model, the levels of ferritin, an inflammatory protein, were increased by exposure to 7keto (Figure 6D), which is in align with previous reports [5,16].

## 3. Discussion

CD74, which plays multiple roles in immunity and inflammation, has been associated with intima-media thickness in subjects free from clinical cardiovascular diseases [9] as well as with infarct size and neurological outcomes in subjects with ischemic stroke [14]. In mice, it has been showed that deficiency of CD74 reduces atherosclerosis [10]. However, it remains unknown whether CD74 is related to human plaque severity and clinical manifestations. This is the first study, to the best of our knowledge, to demonstrate that CD74, particularly surface CD74 expression, increases with the progression of human atherosclerotic plaque. Our findings also show, for the first time, that CD74 is associated with increased levels of apoptosis and ferritin in macrophages, TR, and atherosclerosis-related lipid metabolism.

Advanced atherosclerotic plaques associated with the mechanism of thrombosis contain great amounts of inflammatory cells, including macrophages. The functions of CD74 are involved in multiple inflammatory processes in macrophages. Silencing of CD74 significantly decreased NF-kB activation and MCP-1 secretion induced by IFN-gamma [9]. Our previous results showed that macrophage accumulation is associated with plaque severity and rupture [12,13], and the correlation between CD74 and macrophages, or apoptosis, shown in the present study suggests that CD74 in macrophages may indicate instability of atherosclerotic plaques and can serve as a potential marker in atherosclerotic inflammatory processes. Surface CD74 has been found to specifically participate in inflammatory processes [17,18], and the present study revealed surface CD74 expression in human carotid lesions, notably in the areas with macrophages/foam cells. Moreover, THP-1 macrophages exposed to 7keto showed a significant increase in CD74 levels intracellularly as well on the cell surface. Our study also shows a significant correlation between CD74 and the thrombin receptor. Although the mechanisms behind this are unclear, it has been proposed that inflammation and thrombosis are tightly connected in inflammatory diseases, including atherosclerosis [19]. Increased 7keto levels in atherosclerotic lesions have been reported in previous studies [20,21,22,23]. The oxysterol was mainly detected in areas of advanced atherosclerotic lesions that were rich in macrophages/foam cells. Interestingly, our study showed that areas with high levels of macrophages/foam cells also had high levels of CD74. Moreover, it has been reported that levels of ABCA1, a regulatory protein of cholesterol efflux and a downstream target of the oxysterol nuclear receptor LXR, were decreased in carotid plaques [24,25]. The decreased levels of ABCA1 may lead to a diminished cellular cholesterol efflux followed by enhanced intracellular oxysterol loading, including 7keto, and foam cell formation [24].

High TG and low HDL-C increase the risk of coronary heart disease and stroke through activation of inflammatory molecules, promoting coagulation, and increasing macrophage apoptosis, necrotic core formation, and plaque rupture [15]. HDL has protective effects on atheroma plaque progression through multiple mechanisms, while TG-rich lipoproteins impair HDL function. To the best of our knowledge, there is presently no study reporting on the association between CD74 levels and lipid profiles or statin treatment in atherosclerosis. The inverse correlation between CD74 and anti-atherogenic HDL and statin treatment, and the positive correlation with atherogenic lipid (TG) reported in our study suggests that CD74 levels in atherosclerotic lesions may be modulated by HDL and statin. These findings highlight a potential novel role of CD74 in atherosclerosis-related lipid metabolism; however, further investigations are needed to understand the underlying mechanisms. It has been demonstrated previously that statin treatment in vitro reduced intracellular levels of CD74 in monocytes/macrophages, which supports our findings on the inverse correlation between CD74 and statin treatment [26]. It is well known that one of the cardiovascular benefits of statin is to raise HDL levels [27]. Moreover, it is known that HDL can protect macrophages from oxidized LDL-induced apoptosis by promoting efflux of 7keto via ABCG1 [28]. In the same study, HDL significantly reduced apoptosis induced by 7keto in Abcg1+/+ cells, but not in the Abcg1−/− cells.

Ferritin, an iron storage protein, is considered an inflammatory marker in atherosclerosis [6], as it is mainly a leakage product from damaged cells [29]. We have previously found that overexpression of transferrin receptors and ferritin is related to clinical symptoms and destabilization of human carotid plaques [12]. In another study, we found that exposure to 7-oxycholesterol enlarges the intracellular labile iron pool and induces ferritin, cytosolic accumulation of lipid droplets, and apoptotic macrophage cell death [5]. In the present study, the significant correlation was mostly seen between CD74 and ferritin, however, the causality between the correlation remains unknown. We suggest that ferritin expression may be related to intraplaque hemorrhage because both ferritin and hemoglobin are lesion-dependently accumulated in the human atherosclerotic lesions and ferritin accumulation is related to deposition of hemoglobin and the hemoglobin scavenger receptor [30,31].

In conclusion, our data for the first time suggests that CD74 in apoptotic macrophages is associated with the progression of human atherosclerotic plaques and clinical manifestations. Based on our in vitro and clinical pathology data, we suggest that surface CD74 in apoptotic macrophages and inflammatory ferritin induced by oxidized lipids may contribute to inflammation, thrombosis, and plaque vulnerability in atherosclerosis. Furthermore, CD74 in apoptotic macrophages induced by oxidized lipids may be associated with ferritin levels and lipid metabolism in atherosclerosis.

### Limitation

The present study focuses on the pathology of human carotid atherosclerotic lesions. These carotid lesions were obtained due to either clinical symptoms or as a preventative measure of stroke. Thus, the study is only indicative for possible risks of plaque instability and rupture. The samples sizes are small and a larger prospective study, with a broader patient characteristic profile is needed to confirm the findings of our study.

## 4. Materials and Methods

### 4.1. Collection of Carotid Artery Samples

The atherosclerotic carotid arteries were collected from patients who underwent carotid endarterectomy at Linköping University Hospital. The research protocol was approved by the local ethics committee of Linköping University Hospital (03-499, 2003).

Sixty-one atherosclerotic carotid samples (39 males and 22 females) were included in the present study. Patients with no neurological symptom six months prior to the study were designated as asymptomatic (Asymp, *n* = 8), whereas patients with transitory ischemic attacks, minor stroke, or amaurosis fugax were considered symptomatic (Symp, *n* = 53). Several stroke risk factors, including age, hypertension (defined by hypertension history and diastolic blood pressure ≥ 90 mmHg, all received blood pressure lowering treatment), smoking (defined as regular smoking > 5 years), and diabetes mellitus (defined as regular administration of diabetes medication) were analyzed, which did not show statistical differences between asymptomatic and symptomatic patients (Table S1).

Carotid artery samples were collected immediately post-endarterectomy and fixed in 4% formaldehyde. Three to five cross-sectional segments of each specimen were embedded in paraffin.

### 4.2. Immunohistochemistry

Paraffin-embedded carotid arteries were de-paraffinized in xylene, rehydrated in graded ethanol, and subjected to immunostaining. Immunohistochemistry was performed on serial sections, as described previously [12]. The primary antibodies used were CD74 (Sigma, St Louis, MO, USA), CD68 clone PG-M1 (Dako, Denmark), smooth muscle actin clone 1A4 (Dako, Denmark), ferritin (Dako, Denmark), and thrombin receptor (protease-activated receptor 1, Sigma). The immunoreactions were visualized using the EnVision+/horseradish peroxidase (Dako, Denmark) method and ChemMate EnVision Detection Kit (Dako, Denmark). Control sections without primary antibodies or with non-immune IgG were run for each protocol, resulting in consistently negative results. The slides were counterstained with haematoxylin.

All histological sections were examined under a light microscope, and the images were digitalized with Image Grabber program (Toronto, ON, Canada). The microscope was set on the same parameters used to scan all samples. The randomly digitalized images were analyzed with Adobe Photoshop (v5.5, Adobe Systems Incorporated, San Jose, CA, USA) as described previously [12]. The individual responsible for analysis was blinded to patient information.

### 4.3. Terminal Deoxynucleotidyl-Mediated dUTP Nick End Labeling (TUNEL)

To detect apoptotic cells in the tissue sections, the TUNEL method was employed using an in situ cell death detection kit (Roche Molecular Biochemicals, IN, USA), before being visualized with Fast-red.

### 4.4. Classification of the Plaques

To investigate whether the expression of CD74 is related to plaque progression, all carotid artery samples were classified into three groups based on their morphology and plaque components, as described previously [12]. In brief, the plaques were classified into early and advanced plaques. Early lesions (type 1) have an intact fibrous cap and without necrotic core. Advanced lesions were defined as intact plaques (type 2, with an intact fibrous cap, necrotic core formation and inflammatory cell accumulation) or ruptured plaques (type 3, with a ruptured fibrous cap, often containing a large necrotic core, cholesterol crystals, internal plaque hemorrhage, or thrombosis).

### 4.5. Cell Culture

The THP-1 monocytic cell line was obtained from American Type Culture Collection (ATCC, Rockville, MD, USA) and cultured in RPMI 1640 medium (Invitrogen, Carlsbad, CA, USA) supplemented with 10% fetal bovine serum (Invitrogen, Invitrogen, CA, USA) and 1% penicillin-streptomycin (Invitrogen, Carlsbad, CA, USA) as described previously [32]. The cells were sub-cultured twice a week. THP-1 cells were differentiated into macrophages by incubating with phorbol 12-myristate 13-acetate (50 µM for 24 h). Differentiated macrophages were used for experiments after incubation in normal media for two days. Cholesterol, a structure control, had no effect on CD74 expression and no cytotoxic effect on the cells.

### 4.6. Immunocytochemistry

Cells grown on coverslips were either left untreated or treated with 7keto for 24 h, fixed with 4% PFA for 20 min, and permeabilized with permeabilizing buffer (0.1 g saponin and 5% serum in PBS), followed by incubation with rabbit anti-human CD74 (1:200) for 1 h at room temperature. For the surface staining, cells were incubated with primary antibody for 1 h at room temperature without fixation and permeabilization, and then incubated with goat anti-rabbit Alexa Fluor^®^ 488 (1:200; Invitrogen, Carlsbad, CA, USA) for 1 h at room temperature.

To investigate whether CD74 expression is associated with apoptosis, double immunocytochemistry of CD74 and caspase 3 clone C92-605 (rabbit anti-human monoclonal antibody, BD Biosciences, San Jose, CA, USA) or CD74 and Annexin V (Roche Diagnostics, Mannheim, Germany) was performed. For immunocytochemistry of CD74 and caspase 3, cells were fixed, permeabilized, and incubated with anti-CD74 followed by incubation with Alexa Fluor^®^ 610 (Invitrogen, Carlsbad, CA, USA) for 1 h at room temperature. Then, cells were incubated with FITC conjugated caspase 3 for 1 h at room temperature. For double immunostaining with CD74 and Annexin V, cells were incubated with Annexin V (20 min, 4 °C), followed by fixation with PFA for 15 min. After fixation, cells were incubated with rabbit anti-human CD74 for 1 h at room temperature, before exposure to goat anti-rabbit Alexa Fluor^®^ 610 for 1 h at room temperature.

All immunostained cells were mounted with DAPI-containing mounting media (Vector Laboratories Inc., Burlingame, CA, USA) and analyzed with a Carl Zeiss LSM 700 confocal scanning microscope. The microscope was set on the same parameters used to scan all samples. Images were captured with “Zen lite 2011” software (ZEISS, Oberkochen, Germany), using the 40× oil-immersion objective. The images were analyzed with Adobe Photoshop (v5.5, Adobe Systems Incorporated, San Jose, CA, USA) use the same setting.

### 4.7. Statistics

For immunohistochemistry, continuous data are expressed as mean ± SEM (unless otherwise indicated). Differences were compared by a Kruskal–Wallis test for multiple groups or Mann–Whitney U test for two groups, and Chi-square for comparison of categorical data. Spearman’s correlation test was used to examine the correlation between CD74, CD68, smooth muscle cells (SMCs), apoptosis, ferritin, thrombin receptor, high-density lipoprotein, and triglyceride. The results presented as the Spearman’s correlation coefficient (r). *p* ≤ 0.05 was considered statistically significant.

## Data Availability

The data presented in this study are available from the corresponding author on reasonable request.

## Supplementary Materials

The following supporting information can be downloaded at: https://www.mdpi.com/article/10.3390/metabo12010054/s1, Figure S1: CD74 and TR in CD68 positive macrophages. The immunoreactivity of CD74 and TR in CD68 macrophages in an atherosclerotic region on serial sections of carotid lesions. Note: the similar immuno-staining patterns in CD68, CD74 and TR. Figure S2: Macrophage apoptosis induced by 7keo. The THP1 macrophages were incubated with or without 7keto 28 μM for 24 h. The apoptosis (cellular shrinkage and nuclear condensation) was examined by light microscopy following Wright-Giemsa staining (upper panel). The representative results of the 7keto-treated macrophages analysed by flow cytometry after annexin V/PI staining (lower panel). The values shown in the lower left, lower right, and upper right quadrants of each panel represent the percentage of viable, apoptotic, and post apoptotic necrotic cells, respectively. Table S1: Basic clinical information.
