# Supplementary material for: CD74 in Apoptotic Macrophages Is Associated with Inflammation, Plaque Progression and Clinical Manifestations in Human Atherosclerotic Lesions
Source: Metabolites. 2022 Jan 10;12(1):54. doi: 10.3390/metabo12010054 (PMC8781814; doi:10.3390/metabo12010054)
Supplement: Supplementary file 1 [file metabolites-12-00054-s001.zip › metabolites-1555878-supplementary.pdf]

SUPPLEMENTAL MATERIAL

CD74 in Apoptotic Macrophages Is Associated with Inflammation, Plaque Progression and Clinical Manifestations in Human Atherosclerotic Lesions

Wei Li [1,*], Nargis Sultana [2,3], Linda Yuan [2], Claes Forssell [4] and Xi-Ming Yuan [2]

[1] Obstetrics and Gynecology, Department of Biomedical and Clinical Sciences, Linköping University, 581 85 Linkoping, Sweden
[2] Occupational and Environmental Medicine, Department of Health, Medicine and Caring Sciences, Linköping University, 581 85 Linkoping, Sweden; Nargis.Sultana@regionostergotland.se (N.S.); li8571yu-s@student.lu.se (L.Y.); ximing.yuan@liu.se (X.-M.Y.)
[3] Laboratory medicine, Linköping University Hospital, 581 85 Linkoping, Sweden
[4] Vascular Surgery, Linköping University Hospital, 581 85 Linkoping, Sweden; clafors@gmail.com
* Correspondence: wei.li@liu.se; Tel.: +46-076-161-9736

Supplement Figure S1. CD74 and TR in CD68 positive macrophages. The immunoreactivity of CD74 and TR in CD68 macrophages in an atherosclerotic region on serial sections of carotid lesions. Note: the similar immuno-staining patterns in CD68, CD74 and TR.

Supplementary Figure S2. Macrophage apoptosis induced by 7keo. The THP1 macrophages were incubated with or without 7keto 28μM for 24 h. The apoptosis (cellular shrinkage and nuclear condensation) was examined by light microscopy following Wright-Giemsa staining (upper panel). The representative results of the 7keto-treated macrophages analysed by flow cytometry after annexin V/PI staining (lower panel). The values shown in the lower left, lower right, and upper right quadrants of each panel represent the percentage of viable, apoptotic, and post apoptotic necrotic cells, respectively.

Supplemental Table S1. Basic clinical information.

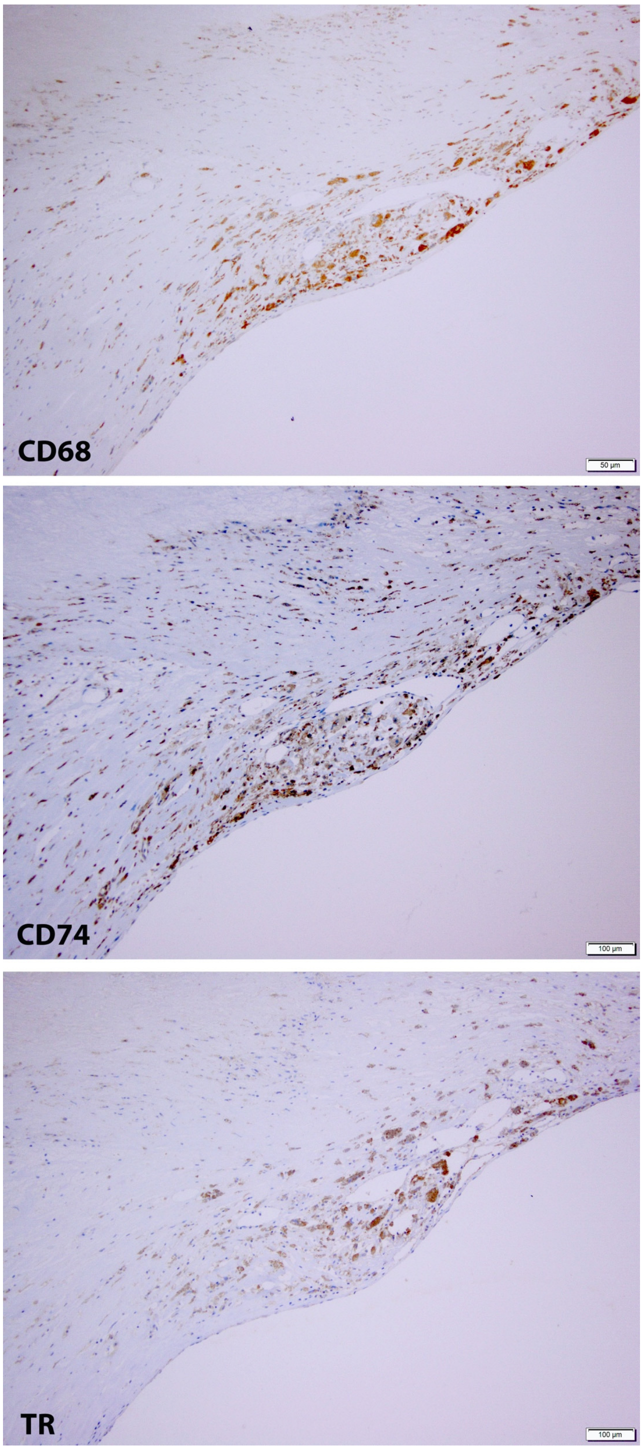

Supplementary Figure S1. CD74 and TR in CD68 positive macrophages. The immunoreactivity of CD74 and TR in CD68 macrophages in an atherosclerotic region on serial sections of carotid lesions. Note: the similar immuno-staining patterns in CD68, CD74 and TR.

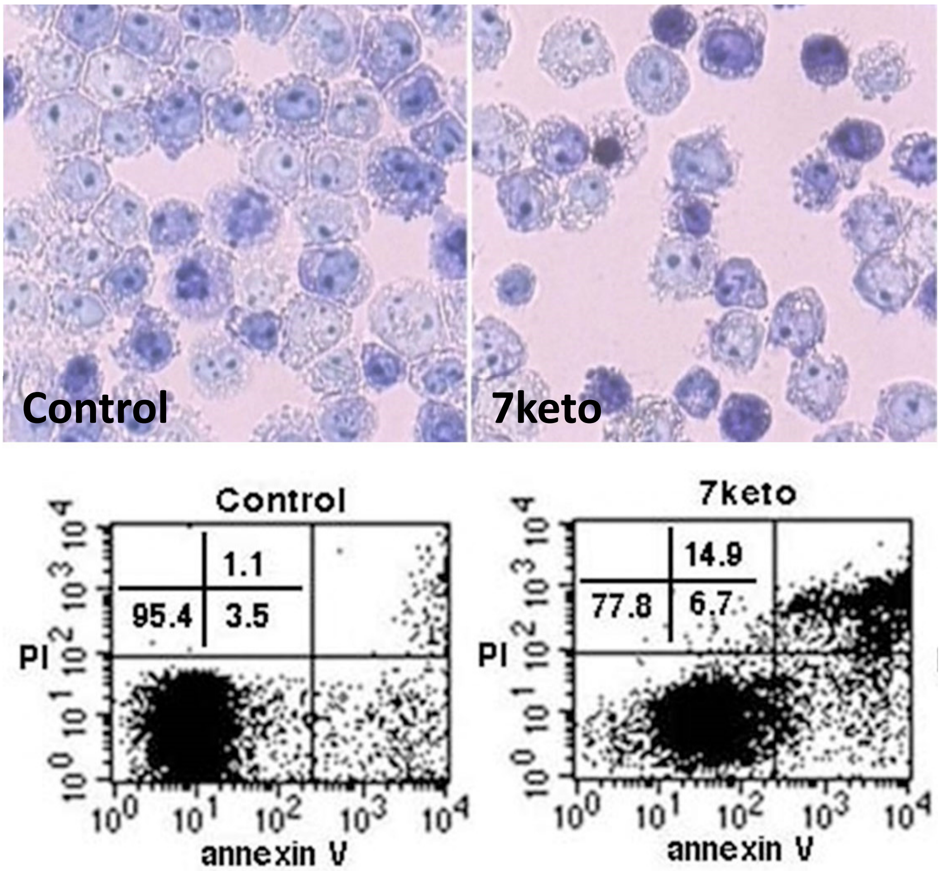

Supplementary Figure S2. Macrophage apoptosis induced by 7keo. The THP1 macrophages were incubated with or without 7keto 28μM for 24 h. The apoptosis (cellular shrinkage and nuclear condensation) was examined by light microscopy following Wright-Giemsa staining (upper panel). The representative results of the 7keto-treated macrophages analysed by flow cytometry after annexin V/PI staining (lower panel). The values shown in the lower left, lower right, and upper right quadrants of each panel represent the percentage of viable, apoptotic, and post apoptotic necrotic cells, respectively.

Supplemental Table S1. Basic clinical information

	Total	Asymptomatic	Symptomatic	
n	61	8	53	p
Age, y ± SE	71.8 ± 1.0	74.4 ± 2.2	71.3 ± 1.1	ns
Sex, male (%, n)	63.9 (39)	62.5 (5)	64.1 (34)	ns
Diabetes mellitus, % (n)	16.4 (10)	25 (2)	15.1 (8)	ns
Hypertesion, % (n)	73.8 (45)	62.5 (5)	75.5 (40)	ns
Smoking, % (n)	39.4 (24)	25 (2)	41.5 (22)	ns
Statin treatment, % (n)	44.3 (27)	62.5 (5)	41.5 (22)	ns
Total cholesterol	5.08 ± 0.20	5.12 ± 0.44	5.07 ± 0.22	ns
LDL	2.77 ± 0.16	2.52 ± 0.55	2.81 ± 0.17	ns
HDL	1.24± 0.08	1.87± 0.38	1.14 ± 0.06	<0.05
TG	2.47 ± 0.39	1.63± 0.34	2.60 ± 0.44	ns
